# Low Molecular Seleno-Aminopolysaccharides Protect the Intestinal Mucosal Barrier of Rats under Weaning Stress

**DOI:** 10.3390/ijms20225727

**Published:** 2019-11-15

**Authors:** Zheng-Shun Wen, Ming Du, Zhen Tang, Tian-Yi Zhou, Zhong-Shan Zhang, Hou-Hui Song, Xing-Wei Xiang, Xin-Yan Han

**Affiliations:** 1 Zhejiang Provincial Engineering Technology Research Center of Marine Biomedical Products, School of Food Science and Pharmaceutics, Zhejiang Ocean University, Zhoushan 316022, China; dm18868009919@163.com (M.D.); tz19920904@126.com (Z.T.); zty17342090270@163.com (T.-Y.Z.); 2 Key Laboratory of Vector Biology and Pathogen Control of Zhejiang Province, Huzhou University, Huzhou 313000, China; 01959@zjhu.edu.cn; 3 College of Animal Science and Technology of Zhejiang A&F University, Key Laboratory of Applied Technology on Green-Eco-Healthy Animal Husbandry of Zhejiang Province, Zhejiang Provincial Engineering Laboratory for Animal Health Inspection and Internet Technology, China-Australian Joint Laboratory for Animal Health Big Data Analytics, Hangzhou 311300, China; songhh@zafu.edu.cn; 4 Department of Food Science and Engineering, Zhejiang University of Technology, Hangzhou 310014, China; xxw11086@126.com; 5 The Key Laboratory of Molecular Animal Nutrition, Ministry of Education. Key Laboratory of Animal Nutrition and Feed Science in East China, Ministry of Agriculture, College of Animal Science, Zhejiang University, 866 Yuhangtang Road, Hangzhou 310058, China; xyhan@zju.edu.cn

**Keywords:** low molecular seleno-aminopolysaccharide, intestinal mucosa, MUC2, Nrf2-Keap1 pathway

## Abstract

Low molecular seleno-aminopolysaccharide (LSA) was synthesized with sodium selenite and low molecular aminopolysaccharide (LA), which is an organic selenium compound. This study is aimed to investigate the protective effect of LSA on the intestinal mucosal barrier in weaning stress rats by detecting the intestinal tissue morphology and function, mucosal thickness and permeability, the structure of MUC2, antioxidant index, the expression level of intracellular transcription factor NF-E2-related factor 2 (Nrf2), and its related factors. The results showed that LSA significantly increased the height of intestinal villi (*p* < 0.05) and increased the thickness of intestinal mucosa and the number of goblet cells, which indicated that LSA has a protective effect on the intestinal mucosal barrier that is damaged by weaning. Moreover, LSA significantly reduced the level of DAO, D-LA, and LPS compared with the weaning group (*p* < 0.05), which indicated that LSA reduced the intestinal damage and permeability of weaning rats. In addition, LSA could increase the number and length of glycans chains and the abundance of acid glycans structures in the MUC2 structure, which indicated that LSA alleviated the changes of intestinal mucus protein structure. LSA significantly increased the levels of GSH-Px, SOD, LDH, and CAT, while it decreased the level of MDA in serum and intestinal tissue, which suggested that LSA significantly enhanced the antioxidant capacity and reduced oxidative stress of weaning rats. RT-PCR results showed that LSA significantly increased the expression level of antioxidant genes (GSH-Px, SOD, Nrf2, HO-1), glycosyltransferase genes (GalNT1, GalNT3, GalNT7) and mucin gene (MUC2) in intestinal mucosa (*p* < 0.05). The results of western blot showed that the LSA activated the Nrf2 signaling pathway by down-regulating the expression of Keap1and up-regulating the expression of Nrf2, and protected the intestinal mucosa from oxidative stress. Overall, LSA could play a protective role in intestinal mucosal barrier of weaning rats by activating the Nrf2 pathway and alleviating the alnormal change of mucin MUC2.

## 1. Introduction

The intestinal tract is likely attacked, invaded, and infected due to direct contact with the external environment, so the intestinal tract plays a very important role in maintaining the health of the body. The clinical onset and severity of intestinal disorders in humans and animals can be profoundly impacted by early life stress [1]. Human infants and neonatal swine experience significant stress in early life that is associated with homeostatic changes related to birth and weaning. Adaptive changes to these early-life stressors are likely contributors to future gastrointestinal disease susceptibility [2]. Weaning stress is easy to result in intestinal mucosal oxidative damage, increase intestinal mucosal damage, and endanger the health of the body. Therefore, how to maintain the healthy state of intestinal mucosa during weaning has become a research hotspot. Studies have previously shown that various stress, especially acute ones, can damage the integrity of the structure and function of the intestinal barrier and increase the permeability of the intestinal mucosa [3]. Animal early weaning stress (EWS) model has been used to study the mechanism of human weaning induced intestinal diseases and related diseases [4]. It has been reported that weaning has a certain disruption effect on intestinal barrier function [5]. The oxidative stress that is caused by weaning leads to the oxidative damage of intestinal mucosa, which is harmful to health. In the healthy state, the production and clearance of intestinal free radicals are in a dynamic balance, to prevent the occurrence and development of diseases. However, oxidative stress is one of the main factors causing various diseases in humans and animals, especially intestinal diseases. Furthermore, the gastrointestinal tract is extremely attacked by excessive free radical, resulting in the abnormal obstruction of intestinal epithelial cell metabolism, damage of intestinal mucosal morphology, increasing permeability, inflammatory response, and intestinal diseases, etc. [6]. Therefore, oxidative stress is the main mechanism that leads to inflammatory bowel disease (IBD), intestinal mucosal infection, crohn′s disease (CD), ulcerative colitis (UC), and colon cancer [7]. Thence, maintaining the healthy status of intestinal mucosa during weaning has become a research hotspot. In addition, as an important translational model for the study of human gastrointestinal diseases, animal models have received increasing attention [8]. Therefore, weaning rats were used as experimental animal models to simulate the weaning process of infants and to study the changes of intestinal barrier function.

Selenium (Se) is an essential micronutrient that combines with cysteine (Sec) to form selenium cysteine (Sec), which plays an antioxidant role [9]. Epidemiological studies have shown that the Se levels in patients with ulcerative colitis (UC) and crohn′s disease (CD) are reduced [10]. This suggests a strong link between selenium and intestinal health. Selenium amino acid, selenium yeast, and selenium polysaccharide are important sources of selenium for human and animals. It has been reported that selenium polysaccharide has higher and more unique biological activities [11]. Therefore, selenium polysaccharide was synthesized with polysaccharide and sodium selenite through synthetic methods [12]. Our laboratory used low molecular weight amino polysaccharide (LA) to synthesize low molecular seleno-aminopolysaccharide (LSA) with sodium selenite. Ultraviolet absorption, infrared absorption spectrum, and NMR characterize the structure of LSA (data not shown). Our previous studies have shown that LSA have low toxicity and higher antioxidant and immunomodulatory activity than selenium and/or LA alone [13,14,15]. However, the effects of selenium-amino polysaccharides on the oxidative damage of intestinal mucosa have been rarely studied. The present study used the intestinal injury model induced by weaning stress to explore the protective effect of LSA on intestinal mucosal barrier in weaning stressed animals and to provide new ideas and methods for regulating intestinal mucosal barrier function in order to evaluate the prevention and treatment of intestinal mucosal injury for LSA.

## 2. Results

### 2.1. Effects of LSA on Intestinal Tissue Morphology of Weaning Rats 

**Figure 1:**
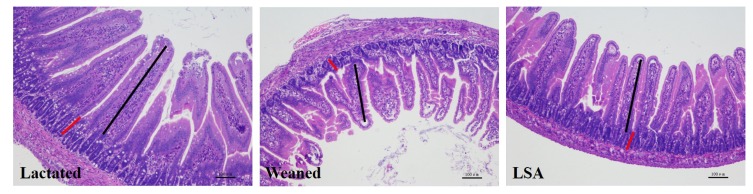
Effects of LSA on jejunal morphology of weaned rats (VH, black line, CD, red line. HE, 100×).

Table 1 and Figure 1 show the results of LSA on intestinal tissue morphology of weaning rats. The results were shown that LSA significantly enhanced the villus height and the ratio of villus height to crypt depth (*p* < 0.05), while there was no significant difference in crypt depth. Moreover, there was no significant difference between the lactated group and the LSA group. In addition, the villus morphology of the LSA group was compacted and complete, while the villus structure of the weaning group was fractured and sparse. The results suggested that LSA could alleviate the intestinal stress reaction that was caused by weaning and protect the integrity of the intestinal structure.

### 2.2. Effects of LSA on Number of Goblet Cells and Intestinal Mucosal Thickness in Weaning Rats 

**Figure 2:**
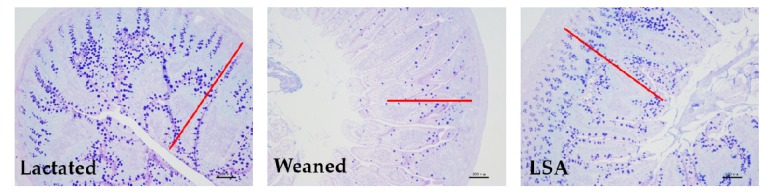
Effects of LSA on number of goblet cells and intestinal mucosal thickness in weaning rats (AB-PAS, the blue dots are goblet cells, 200×).

Figure 2 shows the effects of LSA on a number of goblet cells and intestinal mucosal thickness in weaning rats. AB-PAS staining stains positive goblet cells blue. The results have shown that weaning significantly reduced the number of goblet cells in the ileum as compared with the normal lactation group, which indicated that early weaning seriously affected the intestinal health of juvenile rats. In contrast, the number of goblet cells in the LSA group was significantly increased when compared with the weaning group (*p* < 0.05), and there was no significant difference between the LSA group and the lactated group. In addition, the LSA group had complete intestinal morphology, and more complete and thicker intestinal mucosal layer (red line). It was suggested that LSA could reduce the stress response in the intestines and have a significantly protective effect of intestinal structure integrity of weaning rats.

### 2.3. Effects of LSA on the Level of DAO, D-LA and LPS in Serum

**Figure 3:**
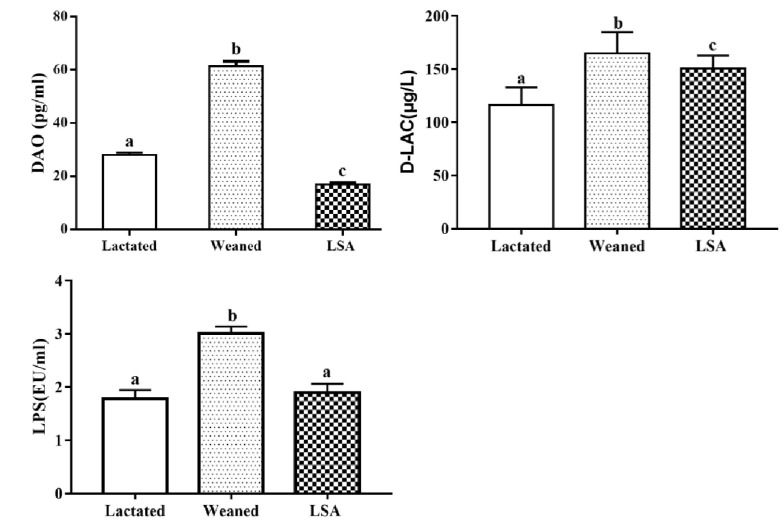
Effects of LSA on the level of mine oxidase (DAO), D-lactic acid (D-LA), and LPS in serum (Bars labeled values with different letters (a, b, c) were significantly different (*p* < 0.05)).

Figure 3 shows the effects of LSA on the level of DAO, D-LA, and LPS in serum. The results shown that weaning could significantly increase the level of serum diamine oxidase (DAO), D-lactic acid (D-LA), and lipopolysaccharide (LPS) (*p* < 0.05). The level of DAO, D-LA, and LPS in LSA group were significantly reduced when compared with the weaning group (*p* < 0.05). Our results suggested that LSA could reduce the intestinal injury and the permeability of intestinal mucosal tissue induced by weaning.

### 2.4. Effects of LSA on the Structure of Intestinal Mucin MUC2

**Figure 4:**
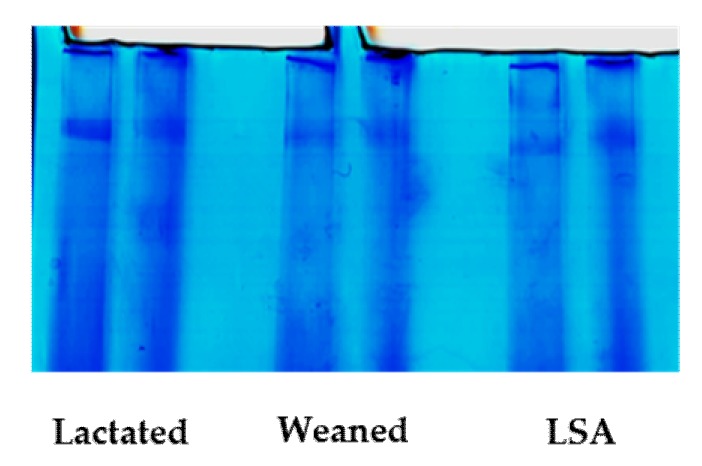
Identification of intestinal mucin MUC2 (Alcian blue staining).

Figure 4 shows the results of SDS-agarose gel electrophoresis of MUC2 of jejunal mucin. As MUC2 is an O-glycoprotein, it can be specifically stained blue by Alcian Blue. Figure 4 showed the alcian blue print, in which the dark blue area is the MUC2 protein band.

**Figure 5:**
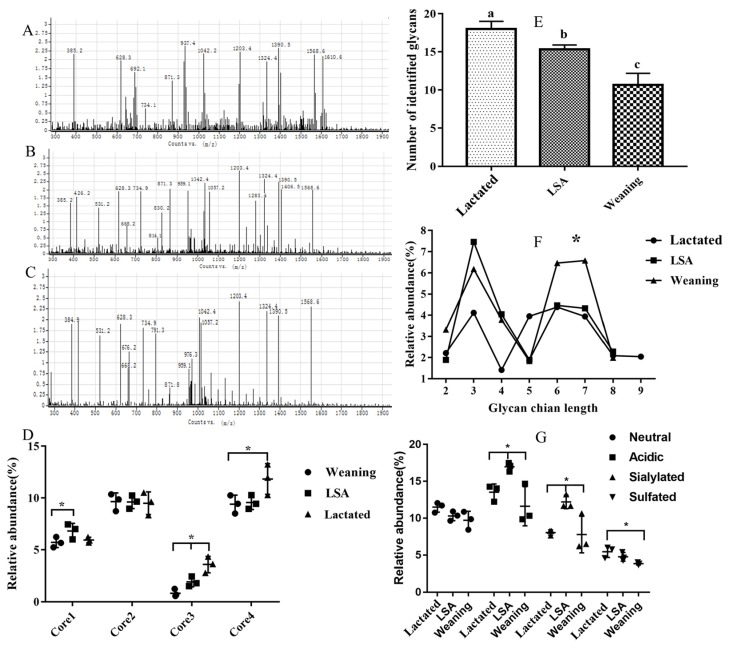
Structure analysis of mucin glycans in different groups. (**A**–**C**) the MUC2 spectra of lactated, weaned and LSA. (**D**) Relative abundance of structures containing core 1–4 present in mucins. (**E**,**F**) number and length of identified glycans. (**G**) Relative abundance of neutral and acidic glycan structures in mucin from different groups. Bars labeled values with different letters (a, b, c) were significantly different (*p* < 0.05). * represents *p* < 0.05.

Figure 5 shows the effects of LSA on the structure of intestinal mucin MUC2. The number of glycans identified in the weaning group was lower than that in the lactated group and the number of glycans in the LSA group was significantly increased when compared with weaning group, which suggested that LSA could significantly alleviate the individual changes of glycans. Moreover, we analysised and compared the abundances of short (<5 monosaccharide residues) and long (>5 monosaccharide residues) glycan chain length identified. The results showed that the chain length of glycans in the lactated group and LSA group was longer than that in the weaning group. In the mucins structure, there was a dominance of structures core 2 and core 4 among three groups, and the abundances of core 4 were higher in the LSA group when compared with the weaning group. In addition, the relative abundances of acidic glycan in mucins from LSA group were higher than that weaning group, including sialylated glycans and sulfated glycans. Our results suggested that LSA could alleviate the changes of intestinal mucin structure caused by weaning.

### 2.5. Effects of LSA on the Antioxidant Indexes in Serum 

**Figure 6:**
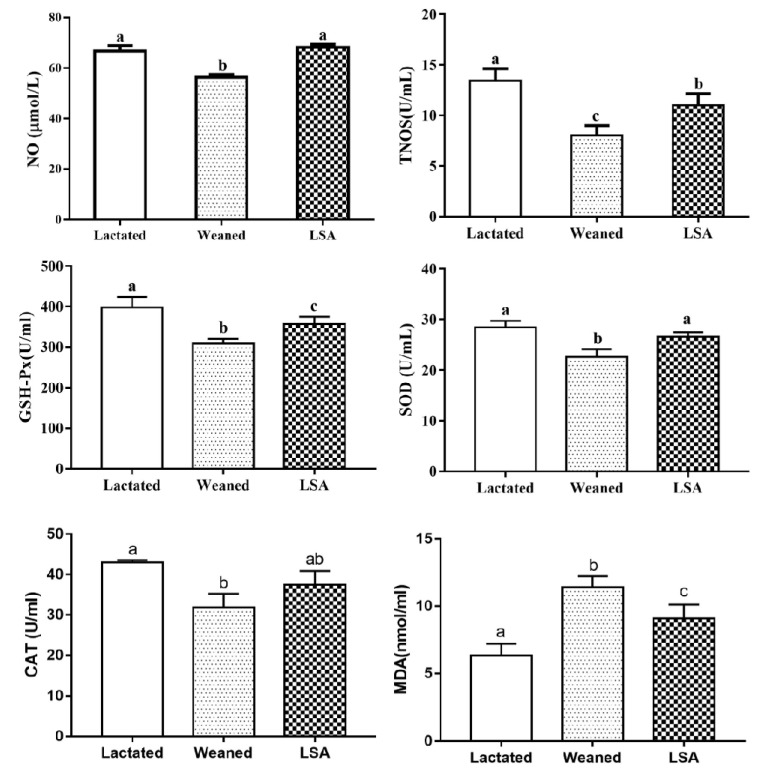
Effects of LSA on the antioxidant indexes in serum (Bars labeled values with different letters (a, b, c) were significantly different (*p* < 0.05)).

Figure 6 shows the effects of LSA on the antioxidant levels in serum of weaning rats. LSA significantly increased the levels of NO, TNOS, SOD, GSH-Px, and CAT in serum, while it significantly decreased the MDA level as compared with the waning group (*p* < 0.05). In contrast, the antioxidant indexes (NO, TNOS, SOD, GSH-Px, and CAT) were significantly descended in weaning group, as well as MDA level was significantly increased (*p* < 0.05). The results suggested that LSA significantly increased the antioxidant capacity and reduced the oxidative stress of weaning rats.

### 2.6. Effects of LSA on the Antioxidant Indexes in Jejunum and Ileum Homogenate 

**Figure 7:**
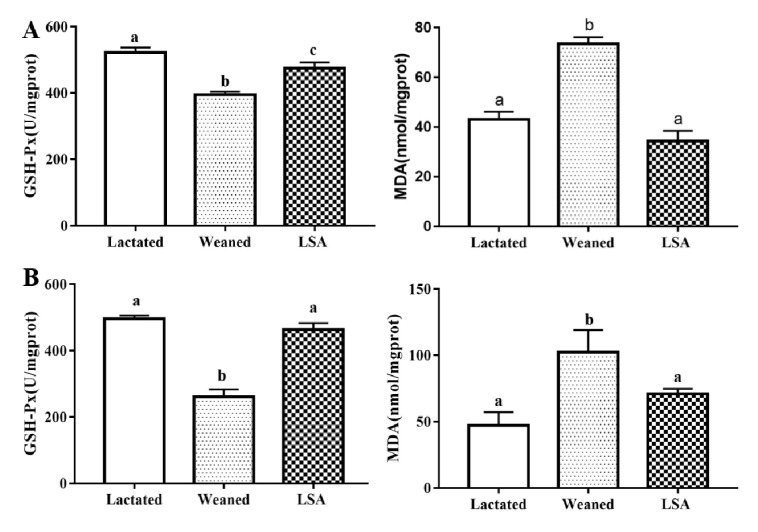
Effects of LSA on antioxidant levels in ileum and colon tissues (**A** ileum and **B** jejunum) (Bars labeled values with different letters (a, b, c) were significantly different (*p* < 0.05)).

Figure 7 shows the effects of LSA on antioxidant levels in jejunum and ileum homogenate (A ileum and B jejunum). When compared with the lactated group, the level of GSH-Px of jejunum and ileum homogenates in the weaning group was significantly reduced (*p* < 0.05), as well as the content level of MDA being significantly increased (*p* < 0.05). In contrast, the level of GSH-Px in LSA group was increased significantly (*p* < 0.05) and the level MDA was significantly reduced (*p* < 0.05). There was no significant difference between the lactated group and LSA group. Those results indicated that LSA significantly enhanced the antioxidant capacity of intestinal tissue homogenate of weaning rats and reduced oxidative damage.

### 2.7. Effects of LSA on the Expression of Antioxidant Genes in Ileum 

**Figure 8:**
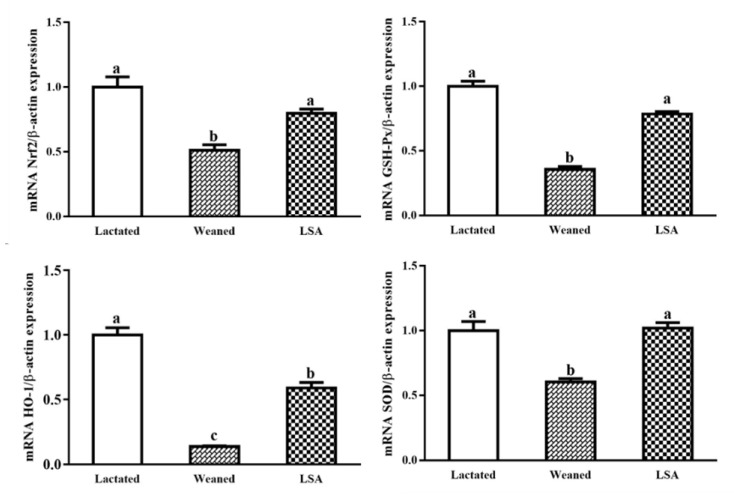
Effects of LSA on the mRNA expression level of antioxidant gene in intestinal mucosa of weaning rats (Bars labeled values with different letters (a, b, c) were significantly different (*p* < 0.05)).

Figure 8 shows the effects of LSA on the expression of antioxidant genes in intestinal mucosa. When compared with the lactated group, GSH-Px SOD Nrf2, and HO-1 gene expression level in the weaning group were decreased significantly (*p* < 0.05), which suggested that weaning significantly reduced antioxidant capacity of the juvenile rat intestinal mucosa and increased oxidative damage that is caused by the weaning stress. In comparison, LSA significantly increased the gene expression level of GSH-Px, SOD, Nrf2, and HO-1 compared with the weaning group (*p* < 0.05). The results suggested that LSA could reduce oxidative damage of intestinal mucosa caused early weaning in rats.

### 2.8. Effect of LSA on mRNA Expression of Glycosyltransferase and Mucin Genes in Intestinal Mucosa of Weaning Rats

**Figure 9:**
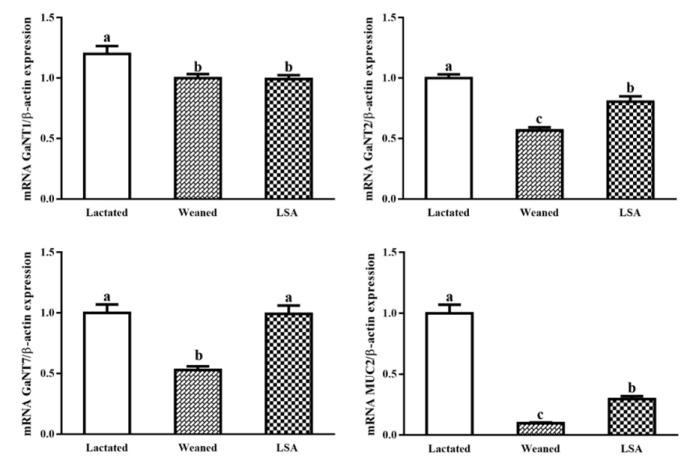
Effect of LSA on mRNA expression of glycosyltransferase and mucin genes in intestinal mucosa of weaning rats (Bars labeled values with different letters (a, b, c) were significantly different (*p* < 0.05)).

Figure 9 shows the effect of LSA on mRNA expression of glycosyltransferase and mucin genes in intestinal mucosa of weaning rats. The mRNA expression level of intestinal glycosyltransferase T1, T2, and T7 in the weaning group were relatively lower than that in the lactated group (*p* < 0.05), which indicated that the expression and secretion of intestinal mucus might be reduced. LSA significantly increased the mRNA expression level of glycosyl transferase T1, T2, and T7 as compared with weaning group (*p* < 0.05), which indicated that the LSA could increase the mucosal glycosyl transferase activity and increase the secretion of mucus. In addition, LSA significantly increased the mRNA expression levels of MUC2 as compared with the weaning group (*p* < 0.05), which suggested that LSA could increase the secretion levels of intestinal MUC2.

### 2.9. Effects of LSA Expression Levels of Transcription Factors in Intestinal Mucosa of Weaning Rats

**Figure 10:**
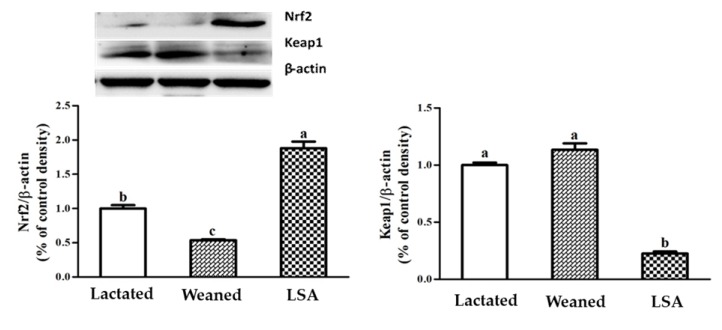
Effect of LSA on expression levels of transcription factors in intestinal mucosa of weaning rats (Bars labeled values with different letters (a, b, c) were significantly different (*p* < 0.05)).

Figure 10 shows the effects of LSA on the expression levels of transcription factors in intestinal mucosa of weaning rats. The results showed that weaning significantly inhibited the Nrf2 protein expression level and enhanced Keap1 protein expression levels as compared with the lactated group (*p* < 0.05). However, LSA significantly increased the Nrf2 protein expression level and inhibited Keap1 protein expression levels when compared with the weaning group (*p* < 0.05). 

## 3. Discussion

Weaning is a stressful time for human infants and animals, and must quickly adapt to sudden psychological, environmental, and nutritional changes. The combined effect of these pressures changes the structure and function of the gastrointestinal tract. Moreover, weaning is associated with intestinal barrier dysfunction, such as increased expression of pro-inflammatory cytokines, reduced enzyme activity, weakened immune response, and intestinal microflora disorder [16]. Hence, the intestinal mucosal barrier plays a very important role in intestinal health and body health after weaning. Villus height (VH), crypt depth, and ratio of villus height to crypt depth (H/D) are indicators of intestinal health, which are often used to evaluate the integrity of intestinal morphology function. In this study, LSA significantly increased the villus height and ratio of villus height to crypt depth (*p* < 0.05), while no significant difference in crypt depth as compared with the weaning group, which suggested that LSA could reduce the intestinal stress response caused by weaning and protect the integrity of the intestinal structure. The intestinal mucosa is covered by a single layer of epithelial cells, including intestinal cells and goblet cells in the villi, stem cells in the crypts, proliferating cells, intestinal endocrine cells, and Paneth cells. The number and distribution of goblet cells play an important role in intestinal mucosal barrier. Meanwhile, intestinal mucosal layer thickness is an important indicator for measuring the function of the mucosal barrier, which refers to the vertical distance from the mucosal epithelium to the mucosal muscle layer [17]. In present study, the effect of LSA on the number and distribution of intestinal goblet cells and the thickness of intestinal mucosa in weaning rats were studied. The results showed that LSA significantly increased the number of goblet cells as compared with the weaned group (*p* < 0.05) and the LSA group had complete intestinal morphology and more complete and thicker intestinal mucosal layer (red line). The results suggested that LSA could reduce the stress response in the intestines and have a significantly protective effect of intestinal structure integrity of weaning rats.

As the first defense barrier of intestinal tract, the small intestinal mucosal barrier plays an important role in protecting potential pathogens from invasion. Intestinal stress that is caused by weaning gave rise to intestinal mucosal barrier injury and pathogenic bacteria invasion. DAO and D-LA levels in serum are important indexes for evaluating the degree of intestinal mucosal injury, and they are negatively correlated with intestinal permeability. When weaning or other stress leads to impaired intestinal permeability, the DAO and D-LA levels were increased. In addition, LPS is a bacteriocin that is secreted by bacteria and it is related with intestinal permeability, which can be used as an indicator to evaluate intestinal mucosal function. In our study, the effects of LSA on intestinal mucosal permeability in weaning rats were studied. LSA significantly reduced the serum levels of diamine oxidase (DAO), D-lactic acid (D-LA), and lipopolysaccharide (LPS) when compared with the weaning group (*p* < 0.05), which indicated that LSA could reduce the injury of intestinal mucosal barrier caused by weaning and significantly reduce intestinal permeability in weaning rats.

Mucin is considered to be a key materiel to the intestinal mucosal barrier, which can be divided into secretory mucin and membrane-bound mucin. Secreted MUC2 forms a layer of mucous on the intestinal surface, which is divided into inner mucus layer and outer mucus layer, and it plays an important role in intestinal health and is the first line of defense against the invasion of harmful pathogens [18]. Mucus lubricates and protects the surface of epithelial cells and it traps viruses and bacteria. Highly glycosylated mucin is the main component of the mucous layer. The inner mucous layer keeps most bacteria at a distance from the epithelial surface, while most bacteria are located in the outer mucous layer [19]. The glycans on the mucin provides a large number of structures on which bacteria can adhere, and the mucin structure also enables the surface of epithelial cells to more tightly adhere to the mucin [20]. In this research, we study the effects of LSA on the structure of intestinal mucin MUC2 in lactation group, weaning group, and LSA group. Twenty-seven glycans have been identified in intestinal mucus, some of which have previously been reported in the intestines of rats, humans, and mice [21]. LSA significantly increased the amount of glycans in the group as compared with the weaning group. Our results indicated that LSA could significantly reduce the individual changes of this glycans. In addition, the abundances of short (< 5 monosaccharide residues) and long (> 5 monosaccharide residues) glycan chain length were identified. The chain length of glycans in the LSA group was longer than that in the weaning group. It has been documented that a small number of patients with ulcerative colitis (UC) have increased the levels of short-chain glycan and sialyl-Tn, which are related to inflammation and the severity of disease [22]. Hence, LSA could improve the changes of mucin structure that are caused by weaning. Glycosylation of MUC2 in the intestine is complex, and there are more than 100 different O-chain glycans on apolipoprotein [23]. These carbohydrates are composed of monosaccharides, which range from 2–12 monomers, and are based on the Core1 to Core5 structures [24]. There were differences in glycosylation patterns in the intestinal tract. The small intestine contained Core2 and Core4 glycosylated glycans with a high degree. Sulfated mucin is generally resistant to the degradation of host protease and bacterial glycosidase, and it is also associated with providing protection to infant intestinal health [25]. Sialic acid terminal residues exert negative charges on mucin and inhibit proteolysis to a certain extent [26]. In our study, there was dominant of structures containing core 2 and core 4 among three groups, and the abundances of core 4 were higher in the LSA group when compared with weaning group. It has been shown in the literature that mice with C1GalT1 and C3GnT knockout have increased susceptibility to colitis, MUC2 level also was decreased, the total glycan chains were shorter, and the microflora was changed [27,28]. Furthermore, previous studies have shown that the activity of sulfatase and sialidase in pathogens has been identified, which are the first step in degrading external mucin, which may cause pathogens to enter and infect epithelial cells, causing inflammatory reactions and leading to intestinal mucosal injury [29]. The structure of acidic glycans and the level of salivary acidic glycans in LSA group were significantly increased as compared with that in the weaning group [30]. Our results suggested that abundant salivary acidification and sulfurization glycans could protect the intestinal mucosal barrier.

Our body is constantly exposed to dangerous and infectious pathogens, and the mucous layer in the enteric cavity separates the external environment from the internal environment of the body. MUC2 and MUC5AC are the main components of intestinal mucus, which are highly hydrated secretory mucins. Their synthesis needs to be initiated by the catalyzed synthesis of 20 UDP-GalNAc family enzymes [31], which is called the *N*-acetylgalactoseaminotransferases (GalNAc-T) family of polypeptides. Previous studies have shown that GalNAc-T1, GalNAc-T2, and GalNAc-T3 were the main initiators of human o-glycosylation [32]. GalNAc-T7 and GalNAc-T10 are subsequent enzymes to the initiation of O-glycation, because they are active against short peptides that have been glycosylated [33]. In this paper, we studied the effects of LSA on the mRNA expression of glycosyltransferase gene and mucin gene in the intestinal mucosa of weaning rats. LSA significantly increased the expression levels of GalNAc-T1, GalNAc-T2, and GalNAc-T7 mRNA of glycosyltransferase in the intestinal mucosa as compared with the weaning group (*p* < 0.05). The results indicated that LSA could increase the activity of glycosyltransferase in the mucosa, thereby increasing the secretion of mucus. In addition, MUC2, as a secretory type, plays an important role in protecting the functional integrity of intestinal mucosal barrier. Compared with the weaning group, LSA significantly increased the mRNA expression level of MUC2 gene (*p* < 0.05). Our results indicated that LSA could increase the expression of MUC2, and thus play a protective role in the intestinal tract.

Under normal physiological circumstances, the generation and elimination of free radicals in the intestinal tract are in a dynamic equilibrium. Excessive free radicals can easily attack intestinal cells, resulting in intestinal oxidative stress. In addition, excessive ROS and RNS, as well as changes in the antioxidant system, may lead to oxidative stress [34]. These mechanisms lead to the occurrence and development of diarrhea, intestinal mucosal infection, UC, CD, and colon cancer [35]. Furthermore, weaning is a stress that is related to intestinal diseases, and oxidative stress that is caused by weaning is an important cause of intestinal mucosal injury [36]. The gastrointestinal organs of the body mainly play the role of antioxidant through antioxidant enzymes (GSH-Px, SOD, CAT, etc.), scavenging free radicals, preventing oxidative damage, and preventing the occurrence and development of diseases [37]. In this study, LSA significantly increased the levels of GSH-Px, SOD, and CAT in serum and intestinal tissue of weaning rats, and decreased the levels of serum and intestinal tissue MDA. In addition, LSA significantly increased the mRNA expression levels of antioxidant genes GSH-Px, SOD, and CAT in ileum mucosa of weaning rats. The results showed that LSA significantly increased the intestinal antioxidant capacity of weaning rats and reduced the oxidative damage.

The activation of Nrf2 induces the production of a large of cytoprotective proteins, such as Heme oxygenase-1 (HO-1), NAD(*p*)H quinine oxidoreductase1 (NQO1), and glutathione transferase (GST) [38]. Nuclear transcription factor Nrf2 belongs to the Cap N Collar (CNC) leucine zipper transcription activator family, which binds to antioxidant response elements (ARE) to induce the expression of antioxidant enzymes and phase II detoxifying enzyme genes [39]. In the present study, LSA significantly increased the mRNA expression levels of Nrf2 gene and its downstream phase II detoxification enzyme HO-1 gene and antioxidant gene (GSH-Px, SOD) in ileum. Our results indicated that LSA played a protective role in activating the Nrf2-ARE signaling pathway. Under normal physiological conditions, Nrf2 binds to Keap1 in large amounts and rapidly degrades in the cytoplasm. When oxidative stress occurs, Nrf2 dissociates from Keap1 and transfers to the nucleus to bind ARE in the promoter region of detoxification enzyme Ⅱ phase enzyme or antioxidant enzymes, which can protect the body from active substances (such as ROS, RNS) and some toxic substances [40]. The activation of Nrf2 signaling pathway is related to antioxidant response, anti-inflammatory response and cell protection, etc. The antioxidant mechanism may be related to the activation of Nrf2 signaling pathway. In order to further determine its action mechanism, western blot method was used to study the translocation Nrf2 and Keap1. Our results showed that LSA significantly inhibited the expression of Keap1 and enhanced the level of the expression of Nrf2. Our findings suggested that the LSA activated the Nrf2 signaling pathways in intestinal mucosa, promoted Nrf2 transcribed into the nucleus, and up-regulated the expression of antioxidant enzymes (GSH-Px, SOD) and stage II detoxification enzymes (HO-1).

## 4. Materials and Methods 

### 4.1. Materials and Reagents

DAO, D-LA, and LPS kits were obtained from Nanjing Senbeijia Biological Technology Co., Ltd. (Nanjing, China). NO, TNOS, SOD, GSH-Px, CAT, and MDA were obtained from Nanjing Jiancheng Bioengineering Institute (Nanjing, China). 2000 DNA Marker, RNA loading buffer, DNA 6×loading buffer, RIPA Lysis buffer, PMSF, BCA kit, BeyoECL Star kit, 5×loading buffer, BSA, non-fat dried milk, TEMED were purchased from Sangon Biotech Co., Ltd. (Shanghai, China). Monoclonal antibodies against β-actin were from Cell Signaling Technology, Inc. (Boston, MA, USA). Antibody against Nrf2 was acquired from Abcam (Cambridge, UK). The antibody against Keap1 was acquired from Santa Cruz Biotechnology, Inc. (Santa Cruz, CA, USA). Alcian Blue, periodic acid schiff were purchased from Beijing solab technology co., Ltd. (Beijing, China). All other reagents are of the highest grade or analytical grade. Our laboratory synthesized the low molecular seleno-aminopolysaccharide (LSA). 

### 4.2. Experimental Animals

All of the procedures of using animals in this study were conformed to measures for the administration of temporary housing for experimental animals that were prepared by the Institutional Animal Care and Use Committee of Zhejiang Ocean University, and the principles of the Zhejiang Ocean University Animal Care and Use Committee approved the animals used in this experiment (SCXK 2013-0016, 28 September, 2017). Male Sprague Dawley (SD) rats at 14 days old weighed 40–60 g rats were purchased from Zhejiang Academy of Medical Sciences. In addition, Rats were adaptively raised in the laboratory for three days. They were housed under same temperature (25 ± 2 °C) and relatively humidity (55 ± 5%) with a 12-h light/12-h dark cycle and kept with free access to food and water. The trial lasted for 14 days. 72 weaning rats were randomly divided into three groups, including Lactated group (lactating for 14 days), Weaned group (weaning for 14 day), and LSA group (7 mg/mL LSA given at a dose of 1 mL/100 g body weight once every day). Each treatment was replicated three times with eight rats per replicate. The LSA was dissolved in 0.01% acetic acid to prepare 7 mg/ mL of LSA solution. At 09:00 every day, the rats were given 1 mL/100 g of body weight by gavage. Before administration, the rats were not allowed to eat, except drinking continuously, and then stopped eating for 3–4 h after gavage.

### 4.3. Sample Collection 

At the end of the experiment, all of the rats were subjected to 12 h fasting, except water, weighted, and injected with 5% pentobarbital sodium solution (50 mg/kg·BW) to anesthetize. Blood was collected from the eyeball by vacuum blood vessel collection, centrifuged at 4 °C (3000 r/min.) for 10 min., and serum was taken. The serum samples were separated into Eppendorf tubes and stored at −80 °C for further analysis. For Histomorphology, the midportion of jejunum were gently rinsed with precooled sterile saline and cleared the contents and fixed at 4% paraformaldehyde solution and store at 4 °C. The ileum mucosa was scraped off with a glass slide and the samples were frozen in liquid nitrogen and stored at −80 °C for analysis.

### 4.4. Histopathological Observation 

At the end of animal experiment, jejunum was taken at about 1 cm. According to the requirements of HE staining, jejunum was quickly repaired to a size of about 1 mm × 1 mm × 1 mm on ice and then fixed pre-cooled 4% glutaraldehyde. The samples were performed in accordance with HE staining procedures.

### 4.5. Thickness of Intestinal Mucosa Layer and the Distribution and Number of Goblet Cells Were Observed by AB-PAS Staining 

The thickness of intestinal mucosa and the distribution and number of goblet cells in the middle segment of jejunum were analyzed by the AB-PAS method. The samples were stained according to the instructions that were provided by the manufacturer of the AB-PAS staining kit, took photos, and analyzed under the Leica inverted microscope.

### 4.6. Determination of Intestinal Permeability by Detecting DAO, D-LA, LPS Levels in Serum

The serum samples were taken out and thawed at room temperature. The levels of diamine oxidase (DAO), D-lactate (D-LA), and lipopolysaccharide (LPS) in serum were detected while using Enzyme-Linked Immunosorbent (ELISA) Assay Kit. The operation process was carried out to the instructions of the kit.

### 4.7. Determination of the Structure of Intestinal Mucin MUC2

The intestinal wall was expanded after the rats were sacrificed under anesthesia. The mucosal surface was lightly rinsed with precooled PBS buffer and mucus was collected in the 1.5 ml EP tube. The structures of glycans were analyzed with Nano–LC/MS. Specific procedures, as previously study described [29]. Briefly, the samples were placed in extraction 6 M GuHCl (5 mM EDTA, 0.01 M NaH_2_PO_4_) and homogenized overnight at 4 °C while using a homogenizer. Subsequently, the samples were centrifuged at 12,300 rpm at 4 °C for 30 min. and removed the supernatants. Pellets were reduced with 50 Mm (final concentration) DTT and stirred at 37 °C for 5 h. Afterwards, the samples were alkylated with 125 Mm iodoacetamide (final concentration) at room temperature and stirred overnight at dark. The sample solution was pooled, dialyzed into water, and lyophilized. SDS–agarose composite gel was used to separate mucins, which was a gradient gel containing agarose (0.5–1% gradient), acrylamide (0–6%), and glycerol (0–10%). The electrophoresis condition was run in the Tris buffer (192 mM boric acid, 1 mM EDTA, 0.1% SDS, pH adjusted to 7.6 with Tris base) on ice in +4 °C at 12 mA/gel overnight (16 h). The gel was wet blotted in a buffer (25 mM Tris, 192 mM glycine, 0.04% SDS, 20% methanol) on ice at 300 mA for 5 h to an Immobilon (PVDF PSQ) membrane. The bands were stained with Alcian blue and then further submitted to β-elimination under reductive conditions (0.1 M NaOH, 1 M KBH4 for 24 h at 45 °C) to release glycans. 

### 4.8. Determination of Antioxidant indexes in Serum 

The levels of NO, TNOS, SOD, GSH-Px, catalase, and MDA in serum were detected with test kits. Specific operation procedures conformed to the manufacturer’s instructions. In addition, the antioxidant activities of intestinal tissue were determinated by detecting the content of GSH-Px and MDA.

### 4.9. Quantitative Real-Time Polymerase Chain Reaction (RT-PCR)

The mRNA expression level of antioxidant genes (GSH-Px, SOD, Nrf2, and HO-1), glycosyltransferase genes (GalNT1, GalNT3, GalNT7), and mucin gene (MUC2) in intestinal mucosa were evaluated by quantitative RT-PCR. Total RNA was extracted from ileum while using precooled TRIzol reagent (15596026, Thermo Fisher Scientific, NY, USA). Measuring the A260/A280 and A260/A230 ratios using nucleic acid protein quantitative assay was determined quality and concentration of the total RNA. PrimeScript™ II 1 st Strand cDNA Synthesis Kit was used to synthesize the first strand cDNA according to the manufacturer′s instructions. Primer 5 software was used to design the primer and we purchased the primer from Sangon Biotech (Shanghai, China) Co., Ltd. The forward and reverse primer sequences were Table 2. Quantitative polymerase chain reaction (PCR) was carried out Applied Biosystems ViiA™ 7 Real-time PCR system (ViiA™ 7 Real-Time PCR System, Thermo Fisher Scientific, Waltham, NY, USA). The total volume of each reaction system is 20 μL (10.0 μL SYBR Green qPCR mix, 2 μL cDNA, 0.4 μL of ROX Ⅱ, 6.8 μL of double distilled water, 0.4 μL of forward primers, and 0.4 μL of reverse primers). The real-time PCR reactions were conformed to the procedure: predenaturation at 95 °C for 30 s, then 40 cycles at 95 °C for 5 s, and at 60 °C for 34 s. After the reaction, the amplification curve and the melting curve were obtained and analyzed to ensure to the specificity of the reaction. The cDNA template that was obtained from reverse transcription was subjected to real-time PCR quantitative fluorescence detection (beta-actin as the internal reference gene), and 2^−∆∆Ct^ calculated the relative mRNA expression level of target genes in each sample.

### 4.10. Western Blot Analysis 

100 mg ileum mucosa samples were grinded fully in liquid nitrogen and collected in a sterile 1.5 mL EP tube. Subsequently, 1 mL RIPA lysis buffer (pre-cooled) was added to sample tube and completely lysed on ice for 15 min. Protein concentration determination was measured by BCA kit. The proper amount of sample was dissolved in sodium lauryl sulfate (SDS) buffer solution and boiled for 5 min at 95–100 °C to denaturate. Afterwards, protein was separated by SDS-PAGE (10%) and subsequently transferred to 0.45 μm polyvinylidene difluoride (PVDF) membranes. The membranes were blocked in 5% non-fat milk for 1 h at room temperature and incubated with primary antibodies at 4 °C overnight. Subsequently, TBST was used to wash the membranes three times for 5 min., each to remove residual primary antibody. Subsequently, the membranes were oscillatingly incubated with secondary antibody (HRP labeled) for 1 h at room temperature according to the instructions. After antibody incubation, TBST was used to wash the membrane three times for 5 min., each to remove residual secondary antibody. ECL reagents were added for chemiluminescent imaging. The bound antibodies were visualized using an enhanced chemiluminescent detection system (Alpha FluorChem FC3, ProteinSimple, SantaClara, CA, USA) and the band density was calculated by AlphaView software. The intensity ratio of proteins examined was relative to β-actin. Each sample was replicated three times. 

### 4.11. Statistical Analysis of Data

All the data were used to undertake statistical analysis with SPSS20.0 software. The data were presented as mean ± standard error of mean (S.E.M.) of three parallel measurements. The statistical significance was analyzed by one-way analysis of variance (ANOVA) and Tukey′s test. *p*-values less than 0.05 were considered to be statistically significant.

## 5. Conclusions

The present study indicated that LSA could increase the height of intestinal villi and increased the thickness of intestinal mucosa and the number of goblet cells. LSA significantly reduced the level of DAO, D-LA, and LPS as compared with the weaning group. In addition, LSA could increase the number and length of glycans chains and the abundance of acid glycans structures in the MUC2 structure. Moreover, LSA significantly enhanced the antioxidant capacity and reduced oxidative stress of weaning rats, while significantly increased the expression level of antioxidant genes (GSH-Px, SOD, Nrf2, HO-1), glycosyltransferase genes (GalNT1, GalNT3, GalNT7), and mucin gene (MUC2) in intestinal mucosa. In addition, LSA could activate the Nrf2 signaling pathway by down-regulating the expression of Keap1 and up-regulating the expression of Nrf2, and protected the intestinal mucosa from the effects of oxidative stress. Collectively, LSA could play a protective role in the intestinal mucosal barrier of weaning stress rats by activating Nrf2 pathway and alleviating alnormal change of mucin MUC2, which suggests that LSA might be used as an active substance for the prevention and management of gastrointestinal disorders by weaning stress in infants.

## Figures and Tables

**Table 1:** Effects of low molecular seleno-aminopolysaccharide (LSA) on jejunal morphology of weaning rats *.

Item	VH (μm)	CD (μm)	VH/CD
Lactated	464.29 ± 14.20 a	124.56 ± 12.30 a	3.72 ± 1.24 a
Weaned	358.25 ± 24.10 b	109.64 ± 8.46 a	3.26 ± 0.89 b
LSA	421.54 ± 18.70 a	113.57 ± 5.68 a	3.76 ± 0.95 a

VH (villus height), CD (crypt depth), LSA (low molecular seleno-aminopolysaccharide) * In the same column, values with different letters (a, b) were significantly different (*p* < 0.05).

**Table 2:** Real-Time Polymerase Chain Reaction (PCR) Primers and Conditions.

Gene	Gene Accession Number	Primer Sequence 5′-3′	PCR Product Size (bp)	Tm
*SOD*	NM_017050.1	F: TCTAAGAAACATGGCGGTCC R: CAGTTAGCAGGCCAGCAGAT	312	60
*GSH-Px*	nm_030826.4	F: CTCTCCGCGGTGGCACAGT R: CCACCACCGGGTCGGACATAC	290	64
*Nrf2*	XM_006234398.3	F: GCTGCCATTAGTCAGTCGCTCTC R: ACCGTGCCTTCAGTGTGCTTC	104	63
*HO-1*	NM_012580.2	F: CGTGCTCGCATGAACACTCT R: GGCGGTCTTAGCCTCTTCTGT	72	61
*GalNT1*	XM_006254471.3	F: ACGACAAGCGTGGTGATT R: CTCGCAGTGAGCATCTAA	291	60
*GalNT2*	NM_001106196.1	F: TGCCAAGCAACAACAAGA R: TTAGCAGCGGACATCGTG	183	60
*GalNT7*	XM_017600074.1	F: CAGAGCCCACTGAGCAGGAT R: TGAATCTGTCATCTCCAGGAGCTA	97	63
*β-actin*	NM_031144.3	F: AGTGTGACGTTGACATCCGTA R: GCCAGAGCAGTAATCTCCTTCT	112	59

F, forward; R, reverse.
